# Acute Ethanol-Disulfiram Reaction Presenting With Hemodynamic Instability: A Case Report

**DOI:** 10.7759/cureus.78735

**Published:** 2025-02-08

**Authors:** Mariana Estrela Santos, Francisca Carmo, João Miranda, Raquel Moura, Janine Resende

**Affiliations:** 1 Internal Medicine, Unidade Local de Saúde Gaia Espinho, Vila Nova de Gaia, PRT

**Keywords:** acetaldehyde toxicity, alcohol dependence, ethanol-disulfiram reaction (edr), hemodynamic instability, supportive management

## Abstract

Ethanol-disulfiram reaction (EDR) is a rare but potentially life-threatening condition characterized by a constellation of symptoms, including flushing, hypotension, tachycardia, nausea, and vomiting. We report the case of a 52-year-old male patient who presented with acute hemodynamic instability after inadvertent alcohol consumption while on disulfiram therapy for alcohol dependence. The patient exhibited signs of shock, including hypotension and hyperlactatemia, but responded promptly to fluid resuscitation and transient vasopressor support. A detailed history confirmed the diagnosis of EDR. This case highlights the importance of recognizing EDR as a differential diagnosis in patients with acute hemodynamic instability and a relevant clinical history. Early identification and supportive management are crucial for favorable outcomes.

## Introduction

Disulfiram is a pharmacologic agent widely used in the treatment of alcohol dependence. It acts by inhibiting the enzyme aldehyde dehydrogenase, leading to the accumulation of acetaldehyde following ethanol consumption. This accumulation triggers an adverse reaction characterized by flushing, nausea, vomiting, tachycardia, and hypotension, collectively referred to as the ethanol-disulfiram reaction (EDR) [1].

The incidence of EDR is underreported, likely due to patient nonadherence and variability in the severity of symptoms. Severe cases, including those with hemodynamic instability and shock, are infrequently documented but underscore the potential seriousness of this condition [2,3]. Diagnostic challenges arise due to its overlap with other causes of acute hypotension such as sepsis, cardiogenic shock, and anaphylaxis [3,4].

In this report, we present a case of severe EDR resulting in hemodynamic instability, discuss the clinical presentation, diagnostic considerations, and management strategies, and review the literature to elucidate the importance of prompt recognition and treatment of this condition.

## Case presentation

A 52-year-old man, employed in the automotive industry, presented to the emergency department with a one-hour history of general malaise, nausea, vomiting, and profuse sweating. His past medical history included chronic alcohol consumption, and he had been prescribed disulfiram as part of his treatment for alcohol dependence. On further questioning, the patient admitted to consuming alcoholic beverages earlier that day.

**Figure 1:**
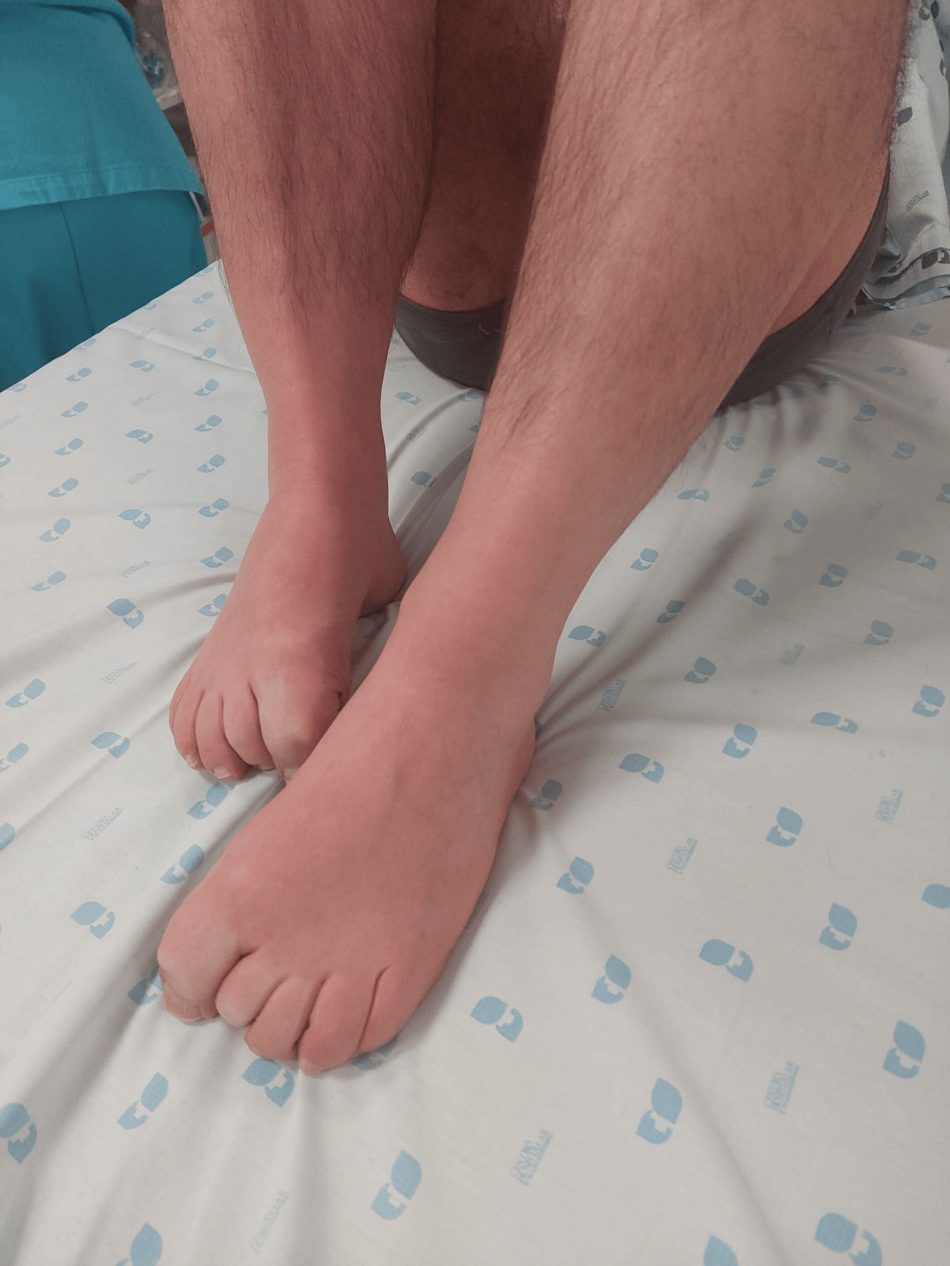
Generalized flushing

On admission, the patient was alert but appeared acutely ill. His vital signs revealed a Glasgow Coma Scale (GCS) score of 14 (E:4, V:4, M:6), tachypnea with a respiratory rate of 35 breaths per minute, hypotension with a blood pressure of 71/42 mmHg, tachycardia at 120 beats per minute and a generalized tremor. Physical examination revealed generalized flushing that blanched with pressure (Figure 1).

**Figure 2:**
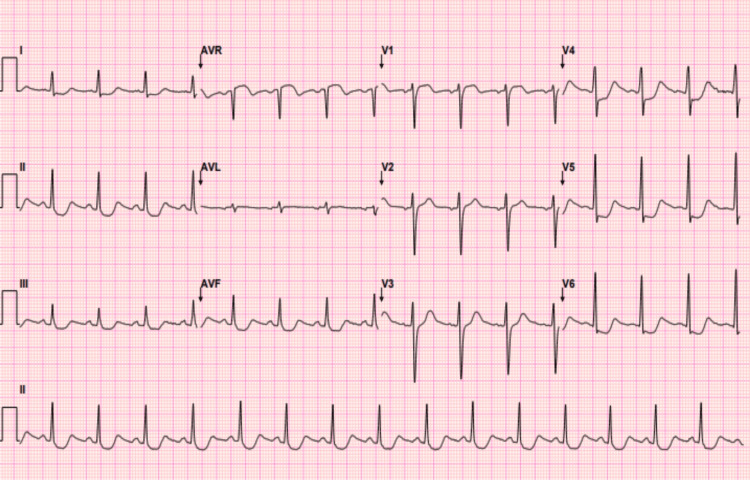
Electrocardiography showed sinus rhythm with ST-segment depression in the anterior leads

Laboratory investigations were significant for an ethanol level of 76.9 mg/dL, hyperlactatemia of 5.5 mmol/L, and respiratory alkalosis with partial pressure of carbon dioxide (pCO2) 27 mmHg. Electrocardiography (ECG) showed sinus rhythm with ST-segment depression in the anterior leads (Figure 2). Bedside transthoracic echocardiography demonstrated hyperdynamic left ventricular systolic function.

The patient was promptly resuscitated with fluid therapy at 30 mL/kg and required transient vasopressor support with norepinephrine (0.02 mcg/kg/minute). Within six hours, vasopressor support was successfully discontinued. Further history and clinical correlation confirmed the diagnosis of EDR.

The patient remained hospitalized for 24 hours for observation, during which all hemodynamic parameters normalized. He was discharged home in stable condition with instructions to discontinue disulfiram and avoid alcohol consumption.

## Discussion

EDR is a well-documented adverse reaction resulting from the pharmacologic interaction between ethanol and disulfiram. The severity of symptoms varies widely, from mild flushing and discomfort to severe hypotension and shock. This variability is influenced by the dose of ethanol consumed, the timing of ingestion relative to disulfiram administration, and individual patient factors [3].

The pathophysiology of EDR involves the accumulation of acetaldehyde, a byproduct of ethanol metabolism, due to disulfiram’s inhibition of aldehyde dehydrogenase. Acetaldehyde exerts systemic effects, including vasodilation, increased capillary permeability, and stimulation of catecholamine release, leading to the clinical manifestations observed [1 ].

Prompt recognition of EDR is critical, as its presentation can mimic other causes of acute hemodynamic instability. Diagnostic clues include a history of recent alcohol consumption, the presence of generalized flushing, and the exclusion of alternative diagnoses. Laboratory findings, such as hyperlactatemia, may reflect tissue hypoperfusion secondary to hypotension [2].

Management of EDR is primarily supportive and includes fluid resuscitation to restore intravascular volume and, if necessary, vasopressor support to maintain adequate perfusion. In severe cases, continuous hemodynamic monitoring and intensive care may be required. Disulfiram should be discontinued immediately, and patients should be counseled regarding the risks of alcohol consumption while on the medication [3-6].

The prognosis for EDR is generally favorable with timely intervention. Most patients recover fully within 24-48 hours, as observed in this case. However, delayed or inadequate treatment can result in significant morbidity and mortality [3,7,8].

## Conclusions

This case underscores the importance of considering EDR in the differential diagnosis of acute hemodynamic instability, particularly in patients with a history of alcohol dependence treated with disulfiram. Early recognition and appropriate supportive management are crucial for preventing adverse outcomes. Physicians should maintain a high index of suspicion for EDR and educate patients on the risks of disulfiram therapy to mitigate the occurrence of such events. Moreover, clinicians must be aware that EDR can also be triggered by a variety of other medications, including antibiotics such as metronidazole or furazolidone, sulfonylureas (e.g., glyburide, tolbutamide, chlorpropamide), certain antifungals, some anticonvulsants (e.g., phenytoin), nitroglycerin or isosorbide nitrates, and several psychiatric agents, to ensure a thorough differential diagnosis and prompt, targeted treatment.

## References

[1] Eriksson CJ (2001). The role of acetaldehyde in the actions of alcohol (update 2000). Alcohol Clin Exp Res.

[2] Mutschler J, Grosshans M, Soyka M, Rösner S (2016). Current findings and mechanisms of action of disulfiram in the treatment of alcohol dependence. Pharmacopsychiatry.

[3] Segher K, Huys L, Desmet T, Steen E, Chys S, Buylaert W, De Paepe P (2020). Recognition of a disulfiram ethanol reaction in the emergency department is not always straightforward. PLoS One.

[4] Guerzoni S, Pellesi L, Pini LA, Caputo F (2018). Drug-drug interactions in the treatment for alcohol use disorders: a comprehensive review. Pharmacol Res.

[5] Ghosh A, Mahintamani T, Balhara YP (2021). Disulfiram ethanol reaction with alcohol-based hand sanitizer: an exploratory study. Alcohol Alcohol.

[6] Lash E, Hack JB (2019). Disulfiram and hypotension in a 53-year-old woman. R I Med J (2013).

[7] Johansson B (1992). A review of the pharmacokinetics and pharmacodynamics of disulfiram and its metabolites. Acta Psychiatr Scand.

[8] Chick J (1999). Safety issues concerning the use of disulfiram in treating alcohol dependence. Drug Saf.

